# The Effect of POSS Type on the Shape Memory Properties of Epoxy-Based Nanocomposites

**DOI:** 10.3390/molecules25184203

**Published:** 2020-09-14

**Authors:** Avraham I. Bram, Irina Gouzman, Asaf Bolker, Noam Eliaz, Ronen Verker

**Affiliations:** 1 Department of Materials Science and Engineering, Tel-Aviv University, Ramat Aviv, Tel Aviv 6997801, Israel; neliaz@tau.ac.il; 2 Space Environment Department, Soreq Nuclear Research Center, Yavne 81800, Israel; irina@soreq.gov.il (I.G.); asaf.bolker@gmail.com (A.B.); rverker@soreq.gov.il (R.V.); 3 Licensing & Safety Office, Israel Atomic Energy Commission, Tel Aviv P.O. Box 7061, Israel

**Keywords:** shape memory polymer (SMP), shape memory effect (SME), epoxy, nanocomposite, polyhedral oligomeric silsesquioxane (POSS)

## Abstract

Thermally activated shape memory polymers (SMPs) can memorize a temporary shape at low temperature and return to their permanent shape at higher temperature. These materials can be used for light and compact space deployment mechanisms. The control of transition temperature and thermomechanical properties of epoxy-based SMPs can be done using functionalized polyhedral oligomeric silsesquioxane (POSS) additives, which are also known to improve the durability to atomic oxygen in the space environment. In this study, the influence of varying amounts of two types of POSS added to epoxy-based SMPs on the shape memory effect (SME) were studied. The first type contained amine groups, whereas the second type contained epoxide groups. The curing conditions were defined using differential scanning calorimetry and glass transition temperature (*T*_g_) measurements. Thermomechanical and SME properties were characterized using dynamic mechanical analysis. It was found that SMPs containing amine-based POSS show higher *T*_g_, better shape fixity and faster recovery speed, while SMPs containing epoxide-based POSS have higher crosslinking density and show superior thermomechanical properties above *T*_g_. This work demonstrates how the *T*_g_ and SME of SMPs can be controlled by the type and amount of POSS in an epoxy-based SMP nanocomposite for future space applications.

## 1. Introduction

The volume and mass budgets of spacecraft are limited. Hence, spacecraft have to be equipped with lightweight, compact, reliable built-in mechanisms for the deployment of antennas, radiators, solar arrays, optical systems, and more. The demand for lighter and smaller deployment systems increased recently due to the growing interest in the so-called “new space” nanosatellites [1]. A class of materials that can be used for lighter and smaller deployment mechanisms is based on shape memory polymers (SMPs) [2,3,4]. SMPs are stimuli-responsive materials that, after being deformed, have the ability to return to their pre-deformed shape by external stimuli, such as light, chemoresponsivity, electric current, electromagnetism, and temperature [5,6,7]. The SMP is comprised of a chemical network that consists of molecular switching segments, i.e., “soft segments” and “hard segments”. Hard segments are the net points that determine the SMP’s permanent shape, while soft segments are able to reduce stiffness upon a particular stimulus, allowing the polymer to be programmed into its temporary shape [8]. Upon exposure to specific stimulus, the molecular switches are triggered, and strain energy that was stored in the temporary shape is released, which consequently leads to shape recovery to the original permanent shape [5]. Since most polymers possess either glass transition or melting temperatures, a typical external stimulus is the temperature [9].

Thermally activated SMPs can memorize a temporary shape at low temperature and return to their permanent shape at higher temperature. The molecular switches are able to reduce their stiffness upon a particular stimulus—in our case, temperature, allowing the polymer to be programmed into its temporary shape and maintain this temporary shape as the SMP is cooled below a certain threshold temperature. The SMP can return to its permanent shape following heating above this threshold temperature. Usually, this threshold temperature is the SMP’s glass transition temperature, *T*_g_ [5]. SMPs can be divided into four classes: covalently crosslinked glassy thermoset networks (class I), such as epoxies; covalently crosslinked semi-crystalline networks (class II), such as semi-crystalline rubbers; physically crosslinked glassy copolymers (class III), such as poly(vinyl acetate) with poly(lactic acid); and physically crosslinked semi-crystalline block copolymers (class IV), such as polyurethane with polyhedral oligomeric silsesquioxane (POSS) hybrid monomer [10].

SMPs have many benefits for space structures compared to traditional mechanisms, such as lightweight, low cost, high specific modulus, high storage energy ability, and predictable recovery upon exposure to external stimulus [11,12]. Furthermore, compared to metallic materials, SMPs have higher strength-to-weight ratio, as well as great flexibility in terms of material design [13]. Due to these advantages, a potential application of SMPs is to replace metallic mechanisms that are used nowadays in spacecraft [2]. One of the most prominent members of the SMP family is based on epoxy resin [14,15,16,17,18]. Epoxy resins are high-performance thermoset polymers with outstanding mechanical properties such as high modulus, high creep resistance, high adhesion strength, good heat resistance, high electrical resistance, and excellent resistance to chemicals [19,20].

In space, qualified SMPs must withstand the harsh space environment. The space environment is characterized by severe conditions such as hypervelocity micro-meteoroids and space debris impacts, ultrahigh vacuum (UHV), ionizing radiation, ultraviolet (UV) and vacuum UV radiation, electrostatic discharge, extreme thermal cycles, and hyperthermal atomic oxygen (AO). The most destructive elements for organic materials selected for low Earth orbit (LEO) space applications are UV radiation and AO [21,22,23]. AO attack may result in surface chemical reactions [24]. Such reactions can cause surface erosion, changes in surface morphology, chemical composition, and both optical and thermo-optical properties. Due to these severe LEO conditions, polymers require AO protection. Usually, AO protection is provided to the spacecraft outer layer by coating it with silicon dioxide (SiO_2_) or indium tin oxide (ITO). However, such protective layers can be damaged due to hypervelocity debris impact, as well as by ground-based handling [24]. A potential solution is to incorporate inorganic constituents such as silica into the polymer backbone [25,26,27,28,29]. POSS is a cage-like silicon oxide-based molecule that contains various organic functional groups [30,31]. Depending on the functional groups, it can be mechanically dispersed in an organic matrix as a molecular filler, without covalent bonding, or it can be covalently linked to the matrix [32,33]. The addition of POSS molecules having the right functional group to be either physically incorporated or chemically bonded to a specific monomer can create a self-passivating hybrid polymer–POSS material with lower AO erosion yield compared to the pristine polymer. This is due to the formation of a passivation layer as the AO oxidizes the SiO_1.5_ POSS into SiO_2_ [28]. Thus, a hybrid organic–inorganic SMP that is based on an epoxy–POSS (EPOSS) nanocomposite material has a great potential for future space applications. The addition of POSS is known also to affect the *T*_g_ and the Young’s modulus of epoxy-based polymers, and it can improve their mechanical, chemical, thermal, and electrical properties [20,32,34,35,36,37,38,39,40,41,42,43,44,45,46,47,48,49,50].

Most of the previous studies of epoxy and POSS have used diglycidyl ether of bisphenol A (DGEBA) resin [36,37,38,40,41,42,43,44,45,46,47,48,49,50]. DGEBA is the most common type of epoxy resin [19]; it was found to have shape memory properties [51]. Prior studies focused on the effect of low concentrations of POSS (less than 10 wt.%) on the nanocomposites’ properties [19,20,39,40,44,45,46,47,48,49,50]. There are only few works on the effect of high concentrations of POSS (up to 50 wt.%) [32,34,35,41,42,43]. The properties of EPOSS nanocomposites depend not only on the POSS type and concentration, but also on the type of epoxy resin, curing agent, curing process, and they directly correlate with the POSS dispersion techniques [19,49,52].

In recent years, studies of the influence of POSS on the SMP properties gained attention in the SMP community [53,54,55,56,57,58,59]. POSS can be used as a chemical or physical crosslinker and, hence, it can enhance the shape recovery and improve the shape recovery rate [53,54,55,56,57,58,59]. However, the influence of POSS on the shape fixity depends on the SMP matrix. For poly(3-caprolactone) and polyurethane-based SMPs, increase of the POSS content caused an increase in the shape fixity due to the growth of switch segments [55,59]. On the other hand, for ethylene propylene diene rubber-based SMPs, increase of the POSS content resulted in a decrease of shape fixity, due to breakage of the crystalline structure and reduction of switching segments [58]. These studies were done on class II and III SMPs [54,55,56,57,58,59]. Epoxy-based class I SMP, such as the one being studied here, is the simplest type of SMP. In general, it has a sharp *T*_g_ and excellent shape recovery due to the nature of its permanent chemical crosslinking [10].

Previous studies concentrated on the effect of addition of a single type of POSS molecule on the shape memory properties of SMPs [53,54,55,56,57,58,59]. Our goal was to study the effect of the type and content of POSS on the shape memory and thermomechanical properties of an epoxy-based SMP, which may be used in the future for deployable space applications. Two types of EPOSS SMPs were developed: the first type contained various amounts (up to 50 wt.%) of POSS with amine functional groups (AM), while the second one contained various amounts (up to 73 wt.%) of epoxide functional groups (EP). These two types of POSS were incorporated separately into an SMP based on DGEBA and amine crosslinker [51]. By doing so, the effect of the POSS type itself on various properties of the SMPs was investigated. Initially, calorimetric measurements using differential scanning calorimetry (DSC) were performed in order to optimize the curing and post-curing processes. Thereafter, the effect of POSS type on the mechanical and thermomechanical properties of the SMPs, such as storage modulus and *T*_g_, were characterized using dynamic mechanical analyzer (DMA). DMA also enabled the calculation of the crosslinking densities of different EPOSS compositions. The effect of the POSS type and content on the shape memory properties was studied quantitatively, in terms of shape recovery, fixity, and recovery speed. Finally, the effect of the shape memory effect (SME) cycle on the bending stress was studied.

## 2. Results and Discussion

### 2.1. Curing and Post-Curing Conditions

In this work, POSS with eight epoxide (EP) functional groups was used to gradually replace an equivalent amount of EPON 826 resin, a rigid aromatic epoxide with two functional groups. Another type of POSS, containing eight secondary amine (AM) functional groups was used to substitute an equivalent amount of Jeffamine D230 crosslinker—a primary aliphatic diamine with four reactive hydrogen atoms. The effect of AM and EP content on the curing and post-curing processes of EPOSS was studied using DSC. The results were used to derive the degree of curing of various compositions.

**Figure 1:**
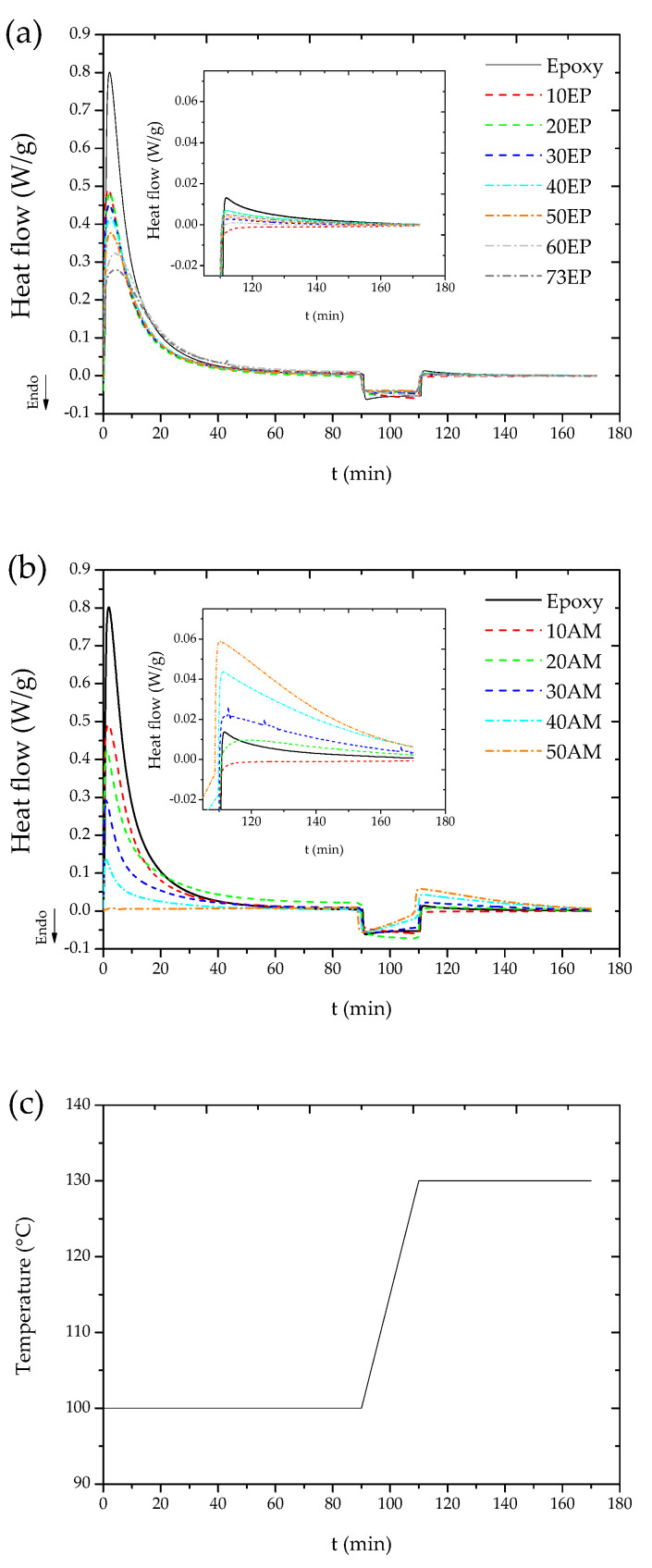
Differential scanning calorimetry (DSC) thermograms of (**a**) epoxide (EP)–EPOSS, (**b**) amine (AM)–EPOSS samples, and (**c**) temperature profiles of the curing (100 °C for 1.5 h) and post-curing (130 °C for 1 h) processes.

DSC thermograms taken during curing and post curing of the epoxide-based EPOSS and amine-based EPOSS formulations, denoted hereafter as EP–EPOSS and AM–EPOSS, respectively, are shown in Figure 1a,b, respectively. The insets show the post-curing stage thermograms of the different compositions. The samples were cured and post cured according to the process shown in Figure 1c and described in Section 3.2. As shown in Figure 1a,b, an increase in POSS content resulted in a reduction of the heat of reaction during curing at 100 °C for both EP–EPOSS and AM–EPOSS samples, indicating a lower degree of curing. The maximal heat flow during the curing stage of EP–EPOSS with the highest POSS content (73 EP, see Table 1) was approximately 34% of the maximal heat flow measured during the curing of the pristine epoxy, 0.27 W/g versus 0.8 W/g, respectively (see Figure 1a). For comparison, during the curing stage of AM–EPOSS with the highest POSS content (50 AM), the heat flow declined to zero (see Figure 1b), indicating that no curing reaction took place at this composition. Higher curing temperatures, up to 160 °C, had a 30% negative effect on the degree of curing of the pristine epoxy. The lower degree of curing may be caused by the diffusion limiting stage becoming dominant earlier compared to the original curing process [60]. Thus, a curing temperature of 100 °C was chosen for all EPOSS compositions.

As shown in the inset of Figure 1a, the higher the EP–POSS content, the lower the measured heat flow during the post-curing stage. For AM–EPOSS samples, an opposite trend can be noticed, i.e., the higher the AM–POSS content, the higher the heat flow measured by DSC (see inset of Figure 1b). While at 100 °C, the 50 AM composition produced no heat flow, at 130 °C, its heat flow was the highest, implying that at this temperature, the AM–POSS forms chemical bonding.

According to Figure 1b, the AM–EPOSS samples had not been cured completely during the curing stage. Moreover, as the curing kinetics of the 50 AM composition is the slowest, determination of the optimal post-curing conditions for the 50 AM ensured appropriate polymerization for all other compositions. Since the curing temperature could not be increased, optimization was performed by increasing the post-curing time. The effect of the post-curing time was assessed by *T*_g_, as it correlates with the crosslinking density; *T*_g_ increases as the crosslinking density increases [61].

**Figure 2:**
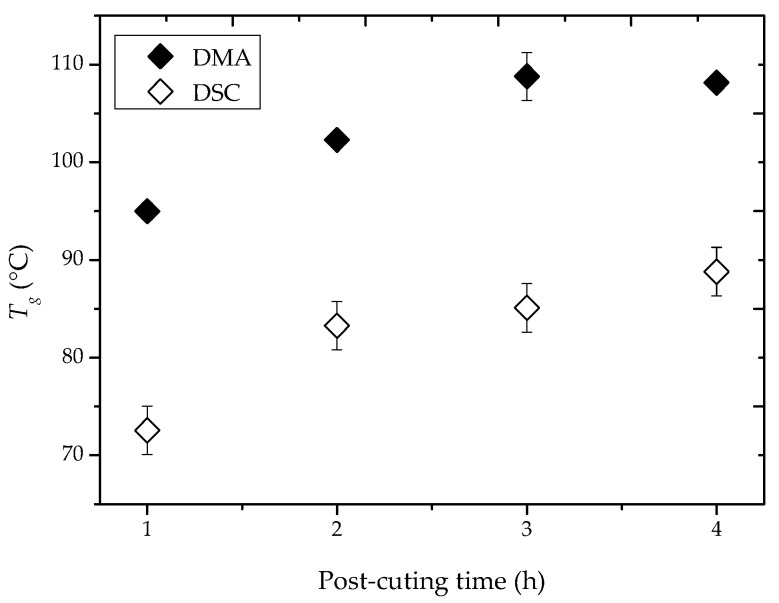
The effect of post-curing time on the glass transition temperatures deduced from both differential scanning calorimetry (DSC) and dynamic mechanical analyzer (DMA) measurements for 50 AM and a post-curing temperature of 130 °C.

*T*_g_ values for 50 AM as a function of post-curing time were measured by both DSC and DMA, as shown in Figure 2. The *T*_g_ values derived from DSC were lower by about 20 °C in comparison to those deduced from DMA, which is in agreement with previous works [38,51]. According to both DSC and DMA measurements, the influence of the post-curing time on *T*_g_ was substantial up to 3 h. *T*_g_ increased from 95 to 108 °C according to DMA or from 72 to 85 °C according to DSC. The addition of another hour had no influence on *T*_g_.

**Figure 3:**
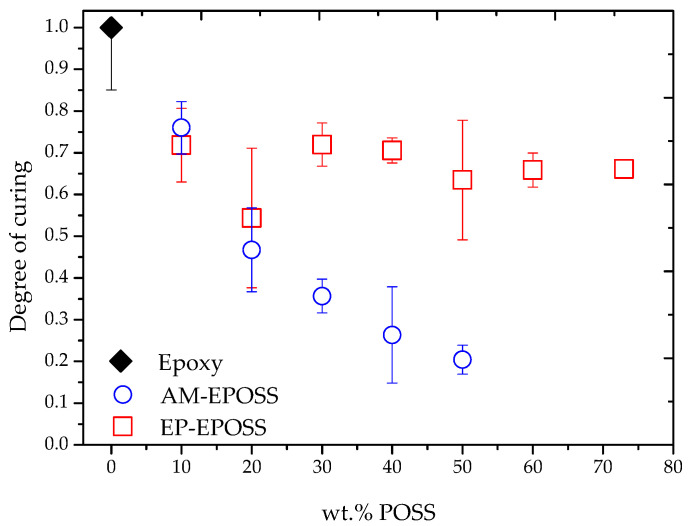
The effect of POSS type and content on the degree of curing of EP–EPOSS and AM–EPOSS.

The degree of curing was extracted from the DSC thermograms for each EPOSS composition, as shown in Figure 3. For both types of POSS, the degree of curing was lower compared to pristine epoxy. When EP–POSS replaced the epoxy resin completely (73 EP), the degree of curing reduced by about 30% compared to the pristine epoxy. It is possible that the EP–POSS molecule has a lower mobility compared to the EPON 826 resin monomers, thus reducing the reaction rate with the crosslinker’s amine groups. This means that although most of the EP–POSS molecules reacted chemically with the resin monomers, the unreacted EP–POSS molecules could be present in the matrix as molecular fillers. When AM–POSS replaced the original crosslinker completely (50 AM), the degree of curing reduced by about 80% compared to pristine epoxy. Compared to the Jeffamine D230 crosslinker, the AM–POSS molecules hardly reacted with the EPON 826 epoxy resin monomers. The sharp reduction in the degree of curing when AM–POSS replaced the Jeffamine D230 crosslinker may be due to lower mobility of the AM–POSS molecules. Therefore, it can be suggested that most of the AM–POSS was dispersed in the organic matrix as a molecular filler, without covalent bonding, whereas only a small amount reacted chemically with the epoxy monomers.

The reduction in the degree of curing may be due to steric interferences resulting from the size of the POSS molecules as well as their functionality. Both the AP–POSS and EP–POSS molecules have eight functional groups, while the original epoxy resin and crosslinker monomers have two and four (two primary and two secondary amines) functional groups, respectively. This means that compared to the original monomers, the steric availability of the functional groups attached to the POSS molecules may be reduced [62]. Moreover, the sharp reduction in the degree of curing in the case of the AM–POSS may be explained by a lower chemical reactivity of phenyl-substituted secondary amine groups attached to the POSS compared to highly reactive primary aliphatic amine groups in the Jeffamine D230 crosslinker [63,64].

### 2.2. Mechanical and Thermomechanical Properties

**Figure 4:**
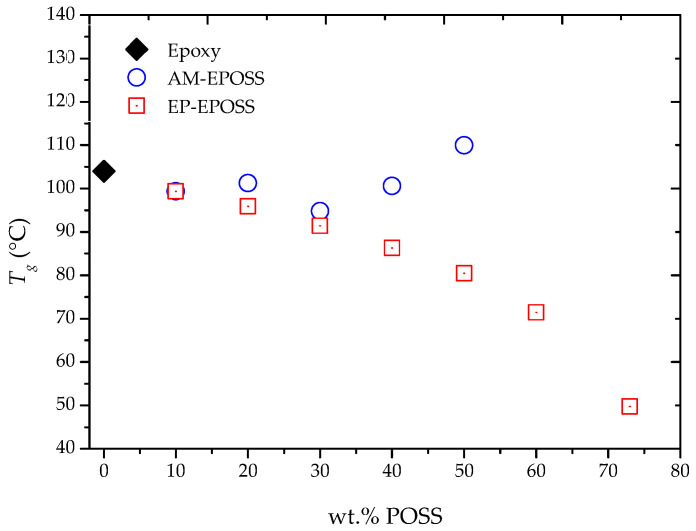
The effect of POSS type and content on the glass transition temperature of EP–EPOSS and AM–EPOSS samples. The error bars are smaller than |±1.6| °C.

Figure 4 summarizes the *T*_g_ values obtained using DMA for different EPOSS compositions. In the case of EP–EPOSS, as the EP–POSS content increased, the *T*_g_ decreased sharply from 104 °C for pristine epoxy to 50 °C for 73 EP. For the AM–EPOSS, *T*_g_ values moderately decreased from 104 to 95 °C as the AM–POSS content increased to 30 wt.%. As the AM–POSS content was further increased from 30 to 50 wt.%, *T*_g_ values increased from 95 to 110 °C.

According to the degree of curing (see Figure 3), most of the EP–POSS molecules are chemically bonded to the crosslinker. Yet, when the EP–POSS content was increased, the *T*_g_ decreased. *T*_g_ is affected by crosslinking density and chain mobility [65]. Therefore, a decrease in *T*_g_ indicates that the addition of EP–POSS leads to an increase of the chain mobility. This phenomenon can be explained by the fraction of POSS molecules that are not chemically crosslinked and are present in the matrix as molecular fillers. Thus, the free volume of the matrix increases to a state where the chains are more flexible and mobile; therefore, *T*_g_ decreases [43].

In the case of AM–EPOSS, the low degree of curing (Figure 3) indicates that most of these molecules were not chemically crosslinked; therefore, *T*_g_ was expected to decrease. However, the measured values are similar to the *T*_g_ of pristine epoxy for most compositions, and they are even slightly higher in the case of 50 AM. A possible explanation is that most of the AM–POSS molecules serve as physical crosslinkers, for example through π–π interactions between phenyl groups of the AM–POSS molecules and bisphenol A groups from the epoxy resin [44,66,67,68]. Physical crosslinkers may cause an increase in the *T*_g_ and storage modulus [53]. Another possible explanation is that the AM–POSS molecules are dispersed as molecular fillers. The higher the density of a filler, the lower the chains’ mobility and the higher the *T*_g_ [69,70].

**Figure 5:**
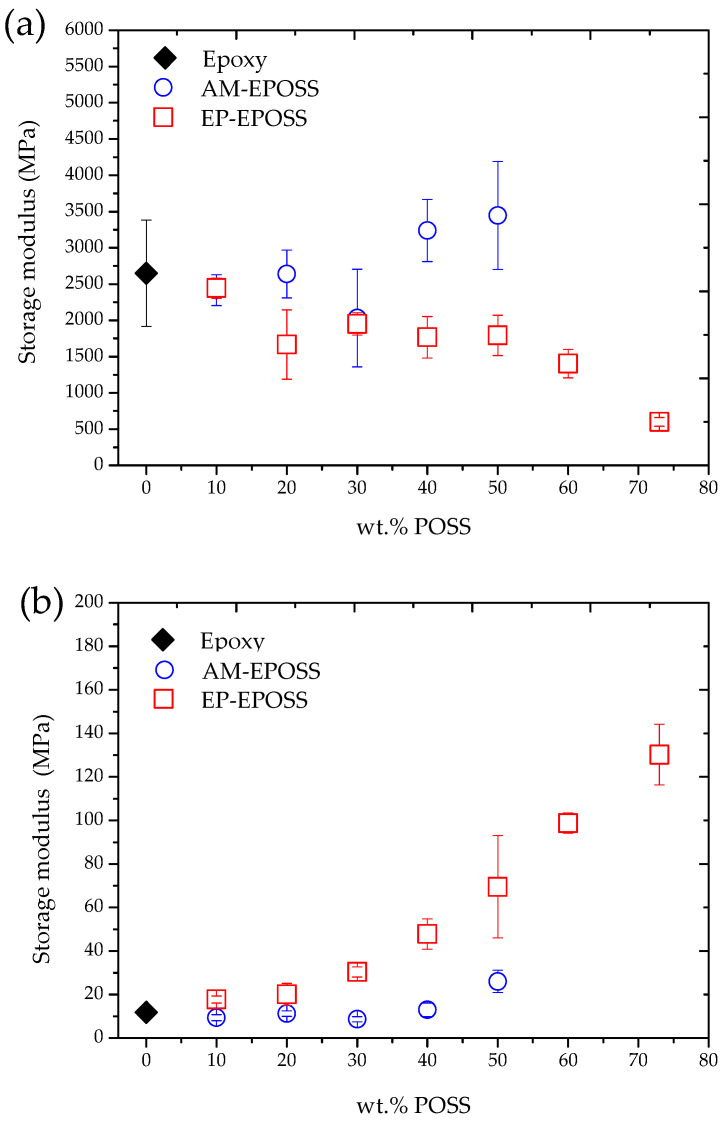
The effect of POSS type and content on the storage modulus of EP–EPOSS and AM–EPOSS shape memory polymers (SMPs) at (**a**) 30 °C and (**b**) 120 °C.

The effect of the POSS type and content on the storage modulus at 30 °C was studied, as shown in Figure 5a. In the case of EP–EPOSS, as the POSS content increased, the storage modulus decreased by up to 77%, from 2650 MPa for pristine epoxy to 600 MPa for 73 EP. For AM–EPOSS, an opposite phenomenon occurred; as the POSS content increased, the storage modulus also increased by up to 30%, from 2650 MPa for pristine epoxy to 3450 MPa for 50 AM. The storage modulus is directly related to the extent of chain mobility, crosslinking density, and filler content. The higher the degree of crosslinking and/or the density of a filler, the lower the chain mobility and the greater the storage modulus [69,70]. According to the results in Figure 5a, at 30 °C, the elasticity of the EP–EPOSS samples was lower than the elasticity of the AM–EPOSS samples. This correlates with the *T*_g_ results shown in Figure 4. The incorporation of EP–POSS might have led to an increase in the free volume of the system and, hence, inefficient packing of the molecular structure. This led to an increase of the chain mobility and a decrease in the storage modulus and material elasticity. It appears that although the degree of curing of AM–EPOSS decreased as the POSS content was increased, the AM–POSS molecules served as efficient physical crosslinkers and/or molecular fillers that decreased chain mobility and, hence, increased the storage modulus and the material elasticity.

A different phenomenon occurred at 120 °C, i.e., above the *T*_g_, for all studied compositions. The storage modulus increased when the POSS content was increased for both types of POSS, see Figure 5b; the storage modulus increased from 10 MPa for pristine epoxy to 30 MPa and 130 MPa for 50 AM and 73 EP, respectively. Moreover, in contrast to the results obtained at 30 °C, at 120 °C, the storage modulus of EP–EPOSS samples was even higher than the storage modulus of the AM–EPOSS samples. It seems that above the *T*_g_, the dominant factor that dictated the polymer elasticity was the crosslinking density, which enabled them to store elastic energy.

**Figure 6:**
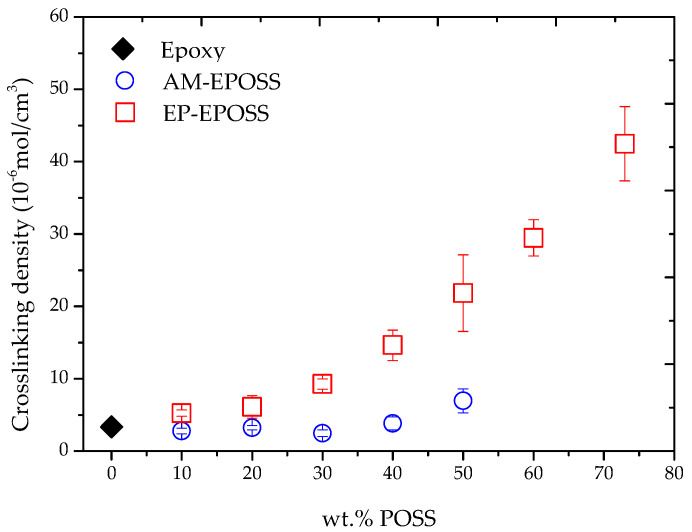
The effect of POSS type and content on the crosslinking density of EP–EPOSS and AM–EPOSS samples.

Figure 6 shows the effect of POSS type and content on the crosslinking density of EP–EPOSS and AM–EPOSS samples, as calculated from the measured storage modulus values. Both types of EPOSS samples have higher crosslinking densities than pristine epoxy, although their degree of curing was lower compared to pristine epoxy (Figure 3). The crosslinking density of the EP–EPOSS increased significantly as more EP–POSS was added to the system, from 3 × 10^−6^ mol/cm^3^ for pristine epoxy to 4.2 × 10^−5^ mol/cm^3^ for 73 EP. Despite AM–EPOSS exhibiting a much lower degree of curing than pristine epoxy, the crosslinking density of these samples hardly changed as POSS was added to the system, excluding the 50 AM. The latter presented a slightly higher crosslinking density than pristine epoxy, 7 × 10^−6^ mol/cm^3^. This means that for each equivalent of Jeffamine D230 chemical crosslinker replaced with AM–POSS, the same amount of physical crosslinking was formed. In addition, the excess of POSS molecules served as molecular fillers in both types of POSS. The crosslinking density was calculated based on the storage modulus in the rubbery region, combining the effects of the chemical and physical crosslinking density as well as the molecular filler density. These results indicate that above *T*_g_, the chemical crosslinking formed by the EP–POSS was more dominant than the physical crosslinking formed by the AM–POSS, leading to higher crosslinking density and storage modulus values at 120 °C.

The difference between the degree of curing and the crosslinking densities arise from the fact that the degree of curing is related solely to heat released during the curing process by chemical reactions, such as polymerization, crosslinking, etc. However, crosslinking density depends not only on chemical reactions, but also on physical interactions between the polymer chains.

### 2.3. Shape Memory Properties

**Figure 7:**
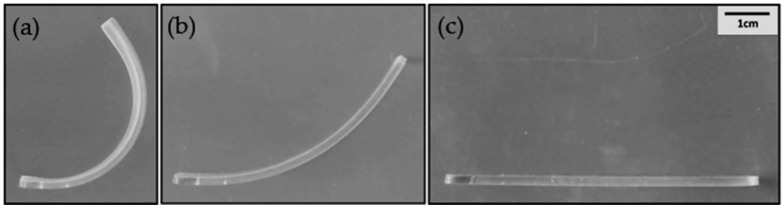
Typical EPOSS SMP in its U-like temporary shape (**a**), during deployment (**b**), and in its final permanent shape (**c**).

**Figure 8:**
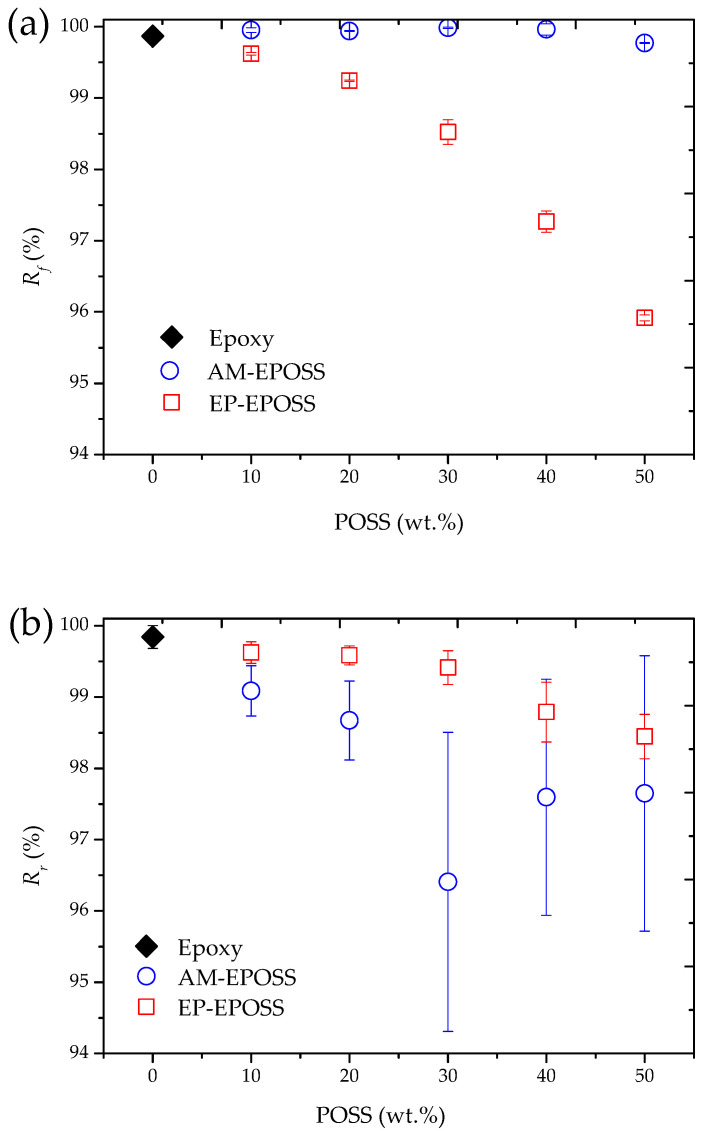
The effect of POSS type and content on (**a**) the shape fixity and (**b**) the shape recovery of EP–EPOSS and AM–EPOSS SMPs.

The SME properties were quantitatively studied during SMP deployment using DMA. Figure 7 presents representative photos of an EPOSS sample in a U-like temporary shape (a), during its deployment stage (b), and at its permanent shape (c). Figure 8 presents the effect of POSS type and content on (a) the shape fixity and (b) the shape recovery of EPOSS SMPs, showing good shape fixity and recovery values. AM–EPOSS SMPs exhibited shape fixity and shape recovery higher than 99% and 96%, respectively. EP–EPOSS SMPs exhibited shape fixity and shape recovery higher than 95% and 98%, respectively. The maximum POSS loading that enabled both types of POSS substantial deformation to a U-like temporary shape and return to the permanent flat shape was 50 wt.%. EP–EPOSS failed during bending at concentrations above 50 wt.% POSS. This can be explained by the high crosslinking density at 60 wt.% and 73 wt.% POSS (Figure 6), and by the high storage modulus values at 120 °C (Figure 5b), which makes the polymer too brittle to be substantially deformed. According to Figure 8a, the shape fixity of EP–EPOSS SMPs decreased from 100% for pristine epoxy to 96% for 50 EP. On the other hand, the shape fixity of the various compositions of AM–EPOSS was around 100%. These results are in accordance with the changes in storage modulus. The storage modulus of EP–EPOSS SMPs at 120 °C was higher than that of AM–EPOSS SMPs. Hence, the various compositions of the EP–EPOSS were less viscous, and they consequently demonstrated lower shape fixity values compared to AM–EPOSS.

During bending (transformation to the temporary shape), tensile stresses are developed on the sample’s convex side, while compressive stresses are developed on the sample’s concave side. The compressive stresses cause the chains and POSS molecules to become closer to each other. On the other hand, tensile stresses cause the chains and POSS molecules to become farther apart. During the deployment of the samples, as they return back to their permanent shape, the chains and POSS molecules reorient to their original position before bending. The difference in the chains and POSS molecules position before bending and after deployment is proportional to the recovery rate value. The higher the recovery rate, the smaller the difference between the original position of the chains and POSS molecules and their position after bending and recovery. The shape recovery decreased when the POSS content was increased for both POSS types. It can be assumed that as the POSS content increased, more POSS molecules were present in the matrix as molecular fillers, which may act as effective chain barriers [71,72]. These molecular fillers increase the hardness of the “soft segments” and decrease the ability of the polymer to return to its permanent shape. The EP–EPOSS SMP compositions show higher recovery values compared to the AM–EPOSS SMPs. This may be explained by a higher degree of curing (see Figure 3) and higher crosslinking density (see Figure 6) of the EP–EPOSS compared to the AM–EPOSS. Thus, EP–EPOSS SMPs have higher net points or hard segments density, which improve the shape recovery [56].

**Figure 9:**
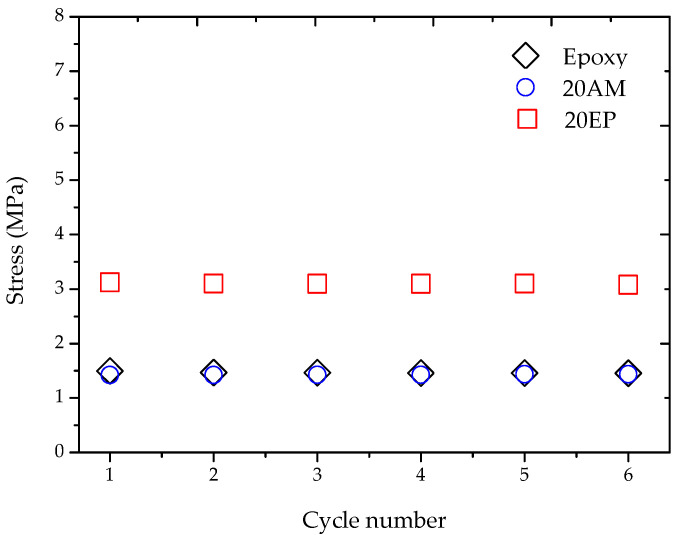
The influence of SME cycles on bending stress of representative EPOSS samples.

Figure 9 shows the bending stress that was applied in order to bend epoxy, 20 AM, and 20 EP samples to their temporary shape, during each of the six consecutive SME cycles. It is observed that the required bending stress remained constant regardless of the SME cycle number: 1.5 MPa for pristine epoxy and 20 AM, and 3.2 MPa for 20 EP. Other EPOSS compositions show the same trend. The representative results of Figure 9 indicate that the mechanical properties of the shape-restored materials are not different from the mechanical properties of the pristine materials.

**Figure 10:**
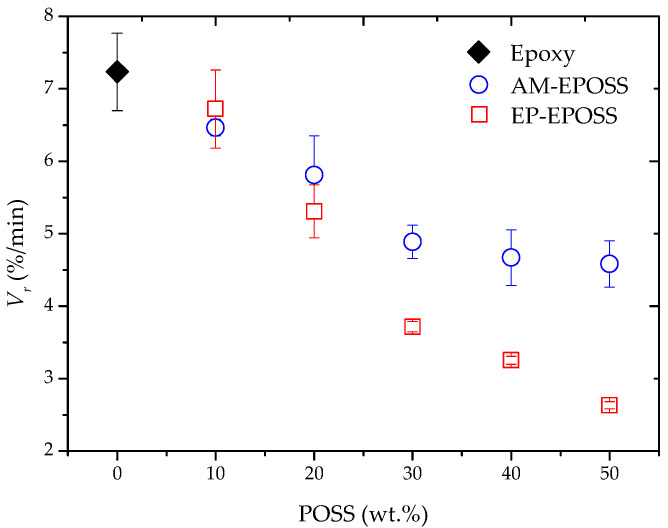
The effect of POSS type and content on the recovery speed of EP–EPOSS and AM–EPOSS SMPs.

Figure 10 shows the influence of POSS type and content on the recovery speed. For both types of EPOSS SMPs, an increase in the POSS content resulted in a decrease in the recovery speed. AM–EPOSS SMPs exhibit a higher recovery speed than EP–EPOSS SMPs. The recovery speed of pristine epoxy is about 7%/min. The incorporation of POSS molecules decreased the speed to 4.5 and 2.5%/min for 50 AM and 50 EP, respectively. The decrease in the recovery speed can be explained by the increase of the free volume due to the presence of the POSS molecules, which in turn decreased the thermal diffusivity [44,73,74]. The increase of the POSS content, either amine- or epoxide-based, also caused an increase in the number of POSS molecules dispersed in the matrix as molecular fillers, which may serve as effective chain barriers that cause the decrease in the recovery speed [71,72]. It is suggested that the lower amount of chemical bonding and higher amount of physical crosslinking resulted in a lower free volume and better thermal properties of the AM–EPOSS compared to EP–EPOSS.

## 3. Materials and Methods

### 3.1. Materials

**Figure 11:**
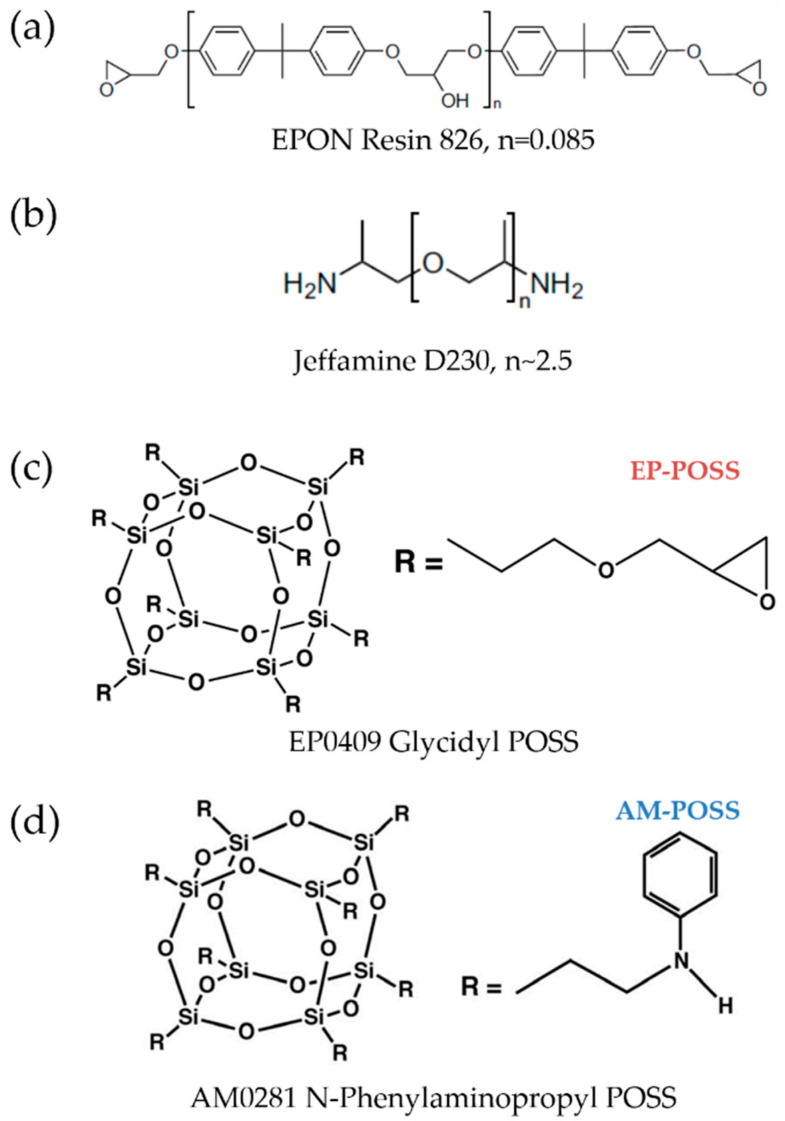
Molecular structures of (**a**) EPON 826 epoxy resin monomer, (**b**) Jeffamine D230 crosslinker, (**c**) EP0409 glycidyl POSS (EP–POSS), and (**d**) AM0281 *N*-phenylaminopropyl POSS (AM–POSS) [51,77,78]. Reprinted from Polymer, Vol. 50, Xie and Rousseau, Facile tailoring of thermal transition temperatures of epoxy shape memory polymers, 1852–1856, Copyright (2009), with permission from Elsevier.

DGEBA epoxy monomer (EPON 826, Momentive, Inc., Waterford, NY, USA) [75] was used as an epoxy resin for the SMP. Poly (propylene glycol) bis (2-aminopropyl) ether (Jeffamine D230, Huntsman, Rotterdam, The Netherlands) [76] was used as a crosslinker agent. N-Phenylaminopropyl POSS^®^ (AM0281, Hybrid Plastics) [77] containing eight amine groups (denoted herein as AM-POSS) and Glycidyl POSS^®^ (EP0409, Hybrid Plastics) [78] containing eight epoxide groups (denoted herein as EP–POSS) were used as alternative additives. The molecular structures of the various EPOSS components are shown in Figure 11.

### 3.2. Epoxy and EPOSS Preparation

Table 1 presents the compositions and designations of EPOSS nanocomposite samples. The AM–POSS or EP–POSS additives substituted the Jeffamine D230 crosslinker or the epoxy EPON 826 resin, respectively, while maintaining a 1:1 molar ratio between the epoxide functional groups and the amine functional groups (both primary and secondary). For example, in order to preserve a 1:1 molar ratio, when adding AM–POSS, the equivalent amount of Jeffamine D230 crosslinker that contains amine functional group was excluded accordingly. A maximum content of the POSS additives was achieved for each of the two types of EPOSS systems by complete replacement of the epoxy resin by the EP–POSS or complete replacement of the Jeffamine D230 crosslinker by the AM–POSS. The maximum POSS weight percent was either 50 or 73 wt.% in AM–POSS and EP–POSS, respectively.

The samples were prepared as follows. The epoxy resin, crosslinker, and POSS additive were weighed and transferred into a glass vial. Then, the vial was heated to 80 °C in order to reduce the epoxy resin and POSS additive viscosities, thus allowing efficient mixing; it was shaken vigorously by a vortex shaker for 1 min at 3000 rpm. Then, the mixture was placed in a vacuum oven preheated to 80 °C. Degassing was performed for 8 min at a pressure of less than 10 Torr. Then, the adhesive was casted in an aluminum mold having 70 × 10 × 1.8 mm and 20 × 3.3 × 1 mm cavities. The curing process of the pristine epoxy was adopted from Xie et al. [51], at 100 °C for 1.5 h, followed by post curing at 130 °C for another 1 h. However, due to the variation in POSS type and content and its effect on the curing process, the post curing was extended to 3 h, as described in Section 2.1.

### 3.3. Characterization Methods

The calorimetric behavior of curing and post-curing processes was studied using DSC 1 STAR^e^ system (Mettler Toledo, Inc., Greifensee, Switzerland). Before each measurement, two-point calibration was performed with indium and zinc. The samples for the DSC measurements were weighed by Sartorius SE2 micro-balance (precision of ±0.1 µg); the sample mass was between 40 and 95 mg. Three samples from each EPOSS composition were placed in aluminum crucibles, and the measurements were done under nitrogen atmosphere flowing at 200 mL/min. The DSC investigation was performed at 100 and at 130 °C, see Figure 1c. A heating rate of 1.5 °C/min was used, simulating the heating rate of the oven used for curing of the SMP samples.

*T*_g_ was determined by the midpoint of the step height of the DSC thermogram while heating the cured samples at a heating rate of 10 °C/min. The degree of curing (*α*) is equal to the ratio of the heat released at time *t, H*(*t*), and the total heat released by the curing and post-curing reaction, *H*_total_ (see Equation (1)) [79]:

(1) $$\alpha \left(t\right)=\;\frac{H\left(t\right)}{H_{\mathrm{total}}}$$

Here, *α* was calculated from the ratio of the total heat released during the curing and post curing of a particular EPOSS composition to the heat released during the curing and post curing of the pristine epoxy resin.

The mechanical and thermomechanical properties of the EPOSS samples were characterized using Q800 model DMA (TA Instruments) using three-point bending fixtures. The experiments were performed at a constant strain of 0.1%, a frequency of 1 Hz, and a temperature ramp rate of 3 °C/min from 30 to 130 °C. The *T*_g_ of each composition was obtained from the maximum value of the tan δ versus the temperature curve of three samples for each EPOSS composition. Tan δ (where δ is the phase lag between stress and strain) is calculated from tan δ = *E*″/*E*′, where *E*″ is the loss modulus and *E*′ is the storage modulus. The crosslinking density, *ρ*, was calculated using the rubber elasticity theory according to Equation (2) [80,81]:

(2) $$\rho \left(\frac{\mathrm{mol}}{{\mathrm{cm}}^{3}}\right)=\;\frac{E^{\prime }}{\phi RT}\;$$

where *E′* (MPa) is measured at *T* = *T*_g_ + 30 °C, ϕ is the front factor (approximated to 1 in the Flory theory), *R* is the gas constant, and *T* is the temperature at *T* = *T*_g_ + 30 °C.

### 3.4. Shape Memory Properties

The Q800 DMA operating in controlled strain mode was used to quantify the SME of the different EPOSS SMPs. Samples having dimensions of 20 × 3.3 × 1 mm were used for this purpose. Six thermal cycles were performed for each EPOSS SMP composition. First, the sample was heated at 5 °C/min to *T* = *T*_g_ + 30 °C (i.e., above the switching temperature) and held isothermally for 10 min. Then, the sample was bended to a temporary shape until a strain of *ε_m_* = 10% was reached. Bending was performed at a strain rate of 5%/min, and the sample was held in its temporary shape isothermally for 20 min. Subsequently, the sample was cooled down to 30 °C at a rate of 3 °C/min while maintaining a constant strain of *ε_m_*. Afterwards, the force was reduced to 0.001 N, and the sample strain (*ε_u_*) was measured upon load removal. In the last stage of the cycle, the sample was heated at 5 °C/min up to a temperature of *T* = *T*_g_ + 30 °C, and the SMP returned to its permanent shape. During this stage, the residual strain (*ε_p_*) was determined, and another heating cycle was performed.

The fixity (*R_f_*) and recovery (*R_r_*) of the SMPs during *N* consecutive thermal cycles were calculated using Equations (3) and (4), respectively. Fixity is the ability of an SMP to preserve its temporary shape after cooling and unloading. Recovery is its ability to return from the temporary shape to its permanent shape after reheating [82].

(3) $$R_{f}\left(N\right)=\;\frac{\varepsilon_{u}\left(N\right)-\varepsilon_{p}\left(N-1\right)}{\varepsilon_{m}\left(N\right)-\varepsilon_{p}\left(N-1\right)}\times 100\approx \;\frac{\varepsilon_{u}\left(N\right)}{\varepsilon_{m}\left(N\right)}\times 100$$

(4) $$R_{r}\left(N\right)=\;\frac{\varepsilon_{u}\left(N\right)-\varepsilon_{p}\left(N\right)}{\varepsilon_{u}\left(N\right)-\varepsilon_{p}\left(N-1\right)}\times 100$$

The recovery process speed (*V_r_*) was determined using Equation (5) [82]:

(5) $$V_{r}\left(\frac{\%}{\min}\right)=\;\frac{0.8R_{rate}\varepsilon_{u}}{T_{90}-T_{10}}\times {\left[\frac{dT}{dt}\right]}_{r}$$

where *T*_10_ and *T*_90_ are the temperatures corresponding to recovery rates (*R*_rate_) of 10% and 90%, respectively, in the recovery rate curve, and $\left[\frac{dT}{dt}\right]$ is the average heating rate during the recovery measurements. The recovery rate, from the temporary shape to the permanent shape, was calculated according to Equation (6) [83]:

(6) $$R_{\mathrm{rate}}\left(T\right)=\;\frac{\varepsilon_{2}-\varepsilon \left(T\right)}{\varepsilon_{2}}$$

where *ε_2_* is the strain of the SMP in its temporary shape and *ε*(*T*) is the strain at a designated temperature (*T*).

## 4. Conclusions

Amine- and epoxide-based epoxy–POSS SMPs (AM–EPOSS and EP–EPOSS, respectively) were prepared with various contents of AM–POSS and EP–POSS. The effects of POSS type and content on the SMPs’ thermomechanical and shape memory properties were studied. The curing degree and the crosslinking density of the nanocomposites were derived from DSC and DMA measurements.

EP–POSS formed mainly chemical crosslinking. The crosslinking density increased with the increase in the EP–POSS content, which was most likely due to the higher functionality of the EP–POSS molecules compared to the original epoxy resin that it replaced. However, the crosslinking density remained constant as more AM–POSS was added to the system, which indicates the prevalence of the physical crosslinking. This physical crosslinking may be formed through π–π interactions between phenyl groups from the AM–POSS molecules and bisphenol A groups from the epoxy resin. The higher functionality of the AM–POSS, compared to the original crosslinker it replaced, also contributed to the conservation of the crosslinking density, although the degree of curing decreased as more AM–POSS was added to the system.

The addition of a significant amount of POSS to the nanocomposites did not compromise excellent shape memory properties. Above *T*_g_, EP–EPOSS SMPs showed higher storage modulus, i.e., elasticity values, compared to AM–EPOSS. As a result, EP–EPOSS exhibited lower shape fixity values than AM–EPOSS SMPs. EP–EPOSS SMPs had higher crosslinking density and, hence, they exhibited a higher shape recovery than AM–EPOSS SMPs. Both types of EPOSS SMPs showed a certain decrease in recovery speed with the increased POSS content. This is due to an increase of the number of POSS molecules dispersed in the matrix as molecular fillers, which served as effective chain barriers. It is also assumed that the recovery speed of AM–EPOSS SMPs is higher than EP–EPOSS due to its chain structure. It is suggested that the lower amount of chemical bonding and higher amount of physical crosslinking in AM–EPOSS resulted in lower free volume and better thermal properties compared to EP–EPOSS.

This work presents novel nanocomposite materials with a high content of POSS and effective means to control the SME triggering temperature. It is shown that by choosing a specific POSS type and content, the mechanical properties, either below or above *T*_g_, can be enhanced. Such nanocomposites with high silicon content and excellent shape memory properties are promising candidates for SMP-based space deployable mechanisms.

## Figures and Tables

**Table 1:** Composition of epoxy–polyhedral oligomeric silsesquioxane (EPOSS) nanocomposite samples.

Sample Designation	wt.% Resin (Epoxide, mol)	wt.% Crosslinker Agent (Amine, mol)	wt.% POSS (Epoxide/Amine, mol)
Pristine epoxy (Epoxy) [51]	75 (1)	25 (1)	0 (0)
10 AM	70 (1)	20 (0.86)	10 (0.14)
20 AM	65 (1)	15 (0.70)	20 (0.30)
30 AM	60 (1)	10 (0.52)	30 (0.48)
40 AM	55 (1)	5 (0.28)	40 (0.72)
50 AM	50 (1)	0 (0)	50 (1)
10 EP	65 (0.86)	25 (1)	10 (0.14)
20 EP	55 (0.72)	25 (1)	20 (0.28)
30 EP	45 (0.58)	25 (1)	30 (0.42)
40 EP	34 (0.44)	26 (1)	40 (0.56)
50 EP	24 (0.30)	26 (1)	50 (0.70)
60 EP	14 (0.18)	26 (1)	60 (0.82)
73 EP	0 (0)	27 (1)	73 (1)
